# Effect of the consumption of brazzein and monellin, two recombinant sweet-tasting proteins, on rat gut microbiota

**DOI:** 10.3389/fnut.2024.1362529

**Published:** 2024-03-21

**Authors:** Vladimir A. Veselovsky, Daria I. Boldyreva, Evgenii I. Olekhnovich, Ksenia M. Klimina, Vladislav V. Babenko, Natalia V. Zakharevich, Andrey K. Larin, Maxim D. Morozov, Polina Y. Zoruk, Petr V. Sergiev, Olga A. Dontsova, Igor V. Maev, Tamara S. Novik, Anatoly A. Kotlobay, Vassili N. Lazarev, Maria A. Lagarkova

**Affiliations:** ^1^Lopukhin Federal Research and Clinical Centre of Physical-Chemical Medicine, Moscow, Russia; ^2^A.N. Belozersky Institute of Physico-Chemical Biology, Lomonosov Moscow State University, Moscow, Russia; ^3^Department of Propaedeutics of Internal Diseases and Gastroenterology, Moscow State University of Medicine and Dentistry, Moscow, Russia; ^4^Scientific Research Centre Pharmbiomed, Moscow, Russia

**Keywords:** microbiome, natural sweetener, sugar substitute, intestinal bacteria, food additive, safety, symbiotic microorganism, microbiota composition

## Abstract

Sweet-tasting proteins (SPs) are proteins of plant origin initially isolated from tropical fruits. They are thousands of times sweeter than sucrose and most artificial sweeteners. SPs are a class of proteins capable of causing a sweet taste sensation in humans when interacting with the T1R2/T1R3 receptor. SP thaumatin has already been introduced in the food industry in some countries. Other SPs, such as monellin and brazzein, are promising products. An important stage in researching SPs, in addition to confirming the absence of toxicity, mutagenicity, oncogenicity, and allergenic effects, is studying their influence on gut microbiota. In this paper we describe changes in the composition of rat gut microbiota after six months of consuming one of two recombinant SPs—brazzein or monellin. A full length 16S gene sequencing method was used for DNA library barcoding. The MaAsLin2 analysis results showed noticeable fluctuations in the relative abundances of *Anaerocella delicata* in brazzein-fed rat microbiota, and of *Anaerutruncus rubiinfantis* in monellin-fed rat microbiota, which, however, did not exceed the standard deviation. The sucrose-fed group was associated with an increase in the relative abundance of *Faecalibaculum rodentium*, which may contribute to obesity. Overall, prolonged consumption of the sweet proteins brazzein and monellin did not significantly change rat microbiota and did not result in the appearance of opportunistic microbiota. This provides additional evidence for the safety of these potential sweeteners.

## Introduction

Sugar is naturally present in all foods containing carbohydrates, including fruits, vegetables, cereals, and dairy products. The human body processes these products slowly, and sucrose provides a stable source of energy for cells. However, sugar is currently used in huge quantities in food production, leading to modern humans consuming ten times more sugar than normal. This habit has resulted in an increase in morbidity due to diseases such as metabolic syndrome, cardiovascular problems, obesity, type 2 diabetes, and more (1, 2). Sweet-testing proteins (SPs), which occur naturally primarily in tropical plants, are considered alternatives to sugar (3). SPs do not have a high caloric content or glycemic index and do not participate in carbohydrate metabolism. Due to their ample sweetness, they can be used in small quantities (4).

The human body is inhabited by trillions of symbiotic microorganisms, most of which are found within the gastrointestinal tract, mainly in the large intestine; they are collectively called the microbiota (5, 6). The composition and activity of gut microbiota (GM) change during human life and are shaped by several factors; most notably, diet and dietary factors are major determinants of GM composition and activity (7). GM changes correlate with health status (8). GM activity in humans includes the degradation of undigested proteins and carbohydrates (sugars, oligosaccharides, peptides, and amino acids), amino acid and monosaccharide fermentation, hydrogen disposal, bile-acid transformation, and vitamin synthesis (9, 10). Any change in the sugar/sweetener profile that we consume redefines the nutrient environment in our gut. How indigenous and exogenous microbes use these environments can result in detrimental or beneficial effects on the host. The dietary and microbiota components that regulate host immunity in the case of metabolic dysfunction and the cellular and molecular mechanisms involved in this process are currently being extensively investigated (11). It was shown that GM protects against developing obesity, metabolic syndrome, and pre-diabetic phenotypes by inducing commensal-specific T-helper 17 (Th17) cells. Eliminating sugar from high-fat diets protected mice from obesity and metabolic syndrome in a manner dependent on commensal-specific Th17 cells. Sugar and group 3 innate lymphoid cells (ILC3) promoted the outgrowth of *Faecalibaculum rodentium* that displaced Th17-inducing microbiota (12).

An important issue that artificial sugar substitute manufacturers have already faced before is the impact of these compounds on GM. The detrimental effect of saccharin and aspartame on microbiota has been demonstrated in some experiments on mice and rats (13). There is also evidence that cyclamate and saccharin have a bacteriostatic effect, which is associated with the inhibition of anaerobic glucose fermentation by microbiota (14).

Another study demonstrates that a violation of the microbiota correlates with the occurrence of glucose tolerance when using saccharin and aspartame. This could be a risk for metabolic syndrome, while using antibiotics in experimental animals, in comparison to using saccharin, eliminated glucose tolerance (15).

As for the SP effect on GM, only thaumatin’s influence on human gut bacteria has been examined (16). Thaumatin at 2 mg/mL was shown to increase the total microbial biomass across all microbiomes and promote *Butyricicoccus*, which is associated with beneficial effects including anti-inflammation in inflammatory bowel disease patients (17). The antimicrobial activity of brazzein in comparison with norfloxacin was also evaluated in *in vitro* experiments (18). The authors concluded that the effect is insignificant because the brazzein concentration that is expected to be used in food is not enough to suppress gut microorganisms. Analysis of the literature data presents a fairly large number of studies done on the effect of artificial sweeteners on microbiota, but no published studies to date on the effect of monellin and brazzein, or any SP other than thaumatin, on GM *ex vivo* (16, 19, 20).

Replacing sugars in the diet with SPs can help reduce the level of obesity and related diseases. Therefore, it is necessary to study the effect of SPs not only on the state of the organism itself, but also on its microbiota. In our recent study, we have shown that long-term intragastric administration of brazzein and monellin did not cause changes in animals’ physiological, biochemical, hematological, morphological, and behavioral parameters (21). In this study, we compare the effect of brazzein and monellin on the bacterial composition of rat GM relative to the effect of sucrose.

## Materials and methods

### Preparation of brazzein and monellin

The recombinant brazzein and monellin preparation was described in Novik et al. (21). Briefly, both recombinant proteins were produced in *Pichia pastoris* yeast. The recombinant brazzein sequence used in this study was identical to the natural one. Recombinant single chain monellin was constructed by fusing natural monellin chain B with chain A via a Gly-Phe linker. The proteins were purified by chromatography, followed by 2 steps of ultrafiltration and lyophilization. Protein identification was performed by expression construct sequencing followed by both PAA gel electrophoresis and mass-spectrometry.

### Animal model

Fecal microbiota was studied on outbred rats 3–4 months old with 180–210 g average body weight. The animals were obtained from a nursery operating according to Good Laboratory Practice (GLP) standards, were bred specifically for this study, and were not involved in any previous experiments. The animals were kept in controlled conditions: at an air temperature of 20–22°C and a relative humidity of 60–70%. The distribution of animals into groups was carried out randomly, using body weight as a criterion. Individual values of body weight did not deviate from the average value of their group by more than 10%.

Two series of experiments were performed. Animals were divided into four groups for each experimental series: two experimental ones and two control ones. Each group consisted of 10 animals. Animals in the experimental groups had aqueous solutions of brazzein or monellin administered intragastrically; animals in the control groups had aqueous solutions of sucrose or distilled water administered intragastrically. Sucrose (Dia-M, Moscow, Russia) was administered intragastrically to control group animals at an equivalent dose (ED), calculated based on the daily norm for humans, 714 mg/kg body weight. The sucrose ED for rats (ED_rat_) was 4.284 mg/kg of body weight. Brazzein is 2000 times sweeter than sucrose; therefore, ED_rat_ for brazzein is 2.14 mg/kg. Monellin is 3000 times sweeter than sucrose; therefore, ED_rat_ for monellin is 1.43 mg/kg of body weight. Animals from the first experimental series received the tested proteins at a dose equivalent to 1 ED_rat_ (2.14 mg/kg of body weight and 1.43 mg/kg of body weight for brazzein and monellin, respectively). Animals from the second experimental series received a dose equivalent to 10 ED_rat_ (21.4 mg/kg of body weight and 14.3 mg/kg of body weight for brazzein and monellin respectively). Proteins and control solutions were administered daily at the same time for 150 days.

### Gut microbiota

Six rats were randomly selected from each of the four experimental groups (receiving tested proteins in doses of 1 ED_rat_ and 10 ED_rat_), a control group which had sucrose administered at a dose of 4.284 mg/kg, and a control group which had distilled water administered. On day 0 (before the start of administration of the tested proteins) and on week 3, 6, 9, 12, and 23 after the start of administration of the tested proteins (once a week), the six selected rats were placed in individual metabolic chambers for 1 h to collect 0.5 g of feces from each rat. The collected fecal samples were frozen at -80°C for subsequent analysis. At these time points, fecal samples were taken from the same rats to monitor the individual change dynamics in the rats’ microbiota during the entire experimental period.

### DNA extraction

Nucleic acids from each of 419 fecal samples were extracted using a MAGNO-sorb (AmpliSens, Russia). 400 μL of PBS was added to each sample and transferred to MagNA Lyser Green Beads (Roche, Switzerland) bead tubes for homogenization on a MagNA Lyser (30 s. 7000 rpm) (Roche, Switzerland). After homogenization, the samples were spun for 1 min at 9000 g. The supernatant was transferred to a new tube and 40 μL proteinase K were added. The samples were then heated for 20 min at 65°C and transferred to a plate where magnetic beads with MagMAX™ Viral/Pathogen Binding Solution (Thermo Fisher Scientific, United States) were added. Further DNA extraction was carried out on a KingFisher™ Purification System (Thermo Fisher Scientific, United States) according to manufacturer’s protocol. The DNA was subsequently quantified on a Qubit 4 fluorometer by an Quant-iT dsDNA BR Assay Kit (Thermo Fisher Scientific, United States).

### 16S rRNA gene amplification and sequencing

The extracted DNA (1–5 ng) was amplified using the 27F (AGAGTTTGATYMTGGCTCAG) and 1492R (GGTTACCTTGTTAYGACTT) primers (Eurogen, Russia) and a Tersus Plus PCR kit (Eurogen, Russia) in a total volume of 25 μL. Amplification was performed on a ProFlex™ PCR System (Thermo Fisher Scientific, United States) with the following PCR conditions: initial denaturation at 95°C for 2 min, then 27 rounds of a three-step temperature cycle (95°C for 1 min, 60°C for 1 min, and 72°C for 3 min), followed by a final extension at 72°C for 2 min and cooling at 4°C. The quality of the amplicons was checked by electrophoresis in 1.5% agarose gel. The final amplicons were purified using KAPA HyperPure Beads (Roche, Switzerland) according to manufacturer’s protocol.

Libraries were prepared according to manufacturer protocol (Ligation sequencing amplicons) with modification. The amplicons were processed with the NEBNext^®^ Ultra™ II End Repair/dA-Tailing Module (New England BioLabs Inc., MA, United States). Barcodes [Native Barcoding Kit 96 (SQK-NBD109.96)] were ligated with Blunt/TA Ligase Master Mil (New England BioLabs Inc., MA, United States). Barcoded libraries were purified using KAPA Pure Beads (Roche, Switzerland). Library concentrations were measured using the Quant-iT dsDNA Assay Kit, High Sensitivity (Thermo Fisher Scientific, United States) and samples were mixed in equimolar amounts. The final adapter (Adapter Mix II Expansion) (Oxford Nanopore Technologies, United Kingdom) was ligated to the pooled library using the NEBNext Quick Ligation Module (New England BioLabs Inc., MA, United States). The prepared DNA library (12 μL) was mixed with 37.5 μL of Sequencing Buffer, 25.5 μL of Loading Beads, loaded onto the R9.4 flow cell (FLO-MIN106; Oxford Nanopore Technologies, United Kingdom), and sequenced on MinION™ Mk1B (Oxford Nanopore Technologies, United Kingdom). MINKNOW software ver. 22.12.7 (Oxford Nanopore Technologies, United Kingdom) was used for data acquisition.

### Bioinformatics analysis

Technical sequences and bases with a quality lower than a 9 Phred score were processed using Porechop^1^ and NanoFilt (22) software. The resulting data were evaluated by the Emu pipeline (23) for taxonomic classification. For batch-effect correction between different sequencing technical runs, ConQur software with default parameters was used (24). Further analyses were performed using the vegan (25) and MicrobiotaProcess packages (26) for GNU/R. Alpha-diversity was estimated using the Shannon index. Beta-diversity was estimated using the Bray–Curtis dissimilarity metric and non-metric multidimensional scaling analysis (NMDS). For the statistical evaluation of observed parameters, the Three-Way analysis of variance with a Benjamini-Hochberg correction for multiple testing, implemented in standard GNU/R statistical instruments, were used. The Permutational Multivariate Analysis of Variance Using Distance Matrices (adonis2) implemented in the vegan package was used for estimating differences in microbial composition of experimental groups. Adonis2-produced *p*-values were processed using the Benjamini-Hochberg correction for multiple testing.

LefSe (27, 28) was used for discovery of microbial taxa significantly linked to experimental groups with parameters first. Test. alpha = 0.01, second. Test. alpha = 0.01. Multivariable association discovery was performed using MaAsLin 2 analysis (29). The MaAsLin 2 analysis was performed with the following parameters: fixed_effects = c(“gender,” “group”), reference = c (c(“group, control”)), random_effects = c(“time_point,” “sourceid”), max_significance = 0.01.

## Results

**Figure 1:**
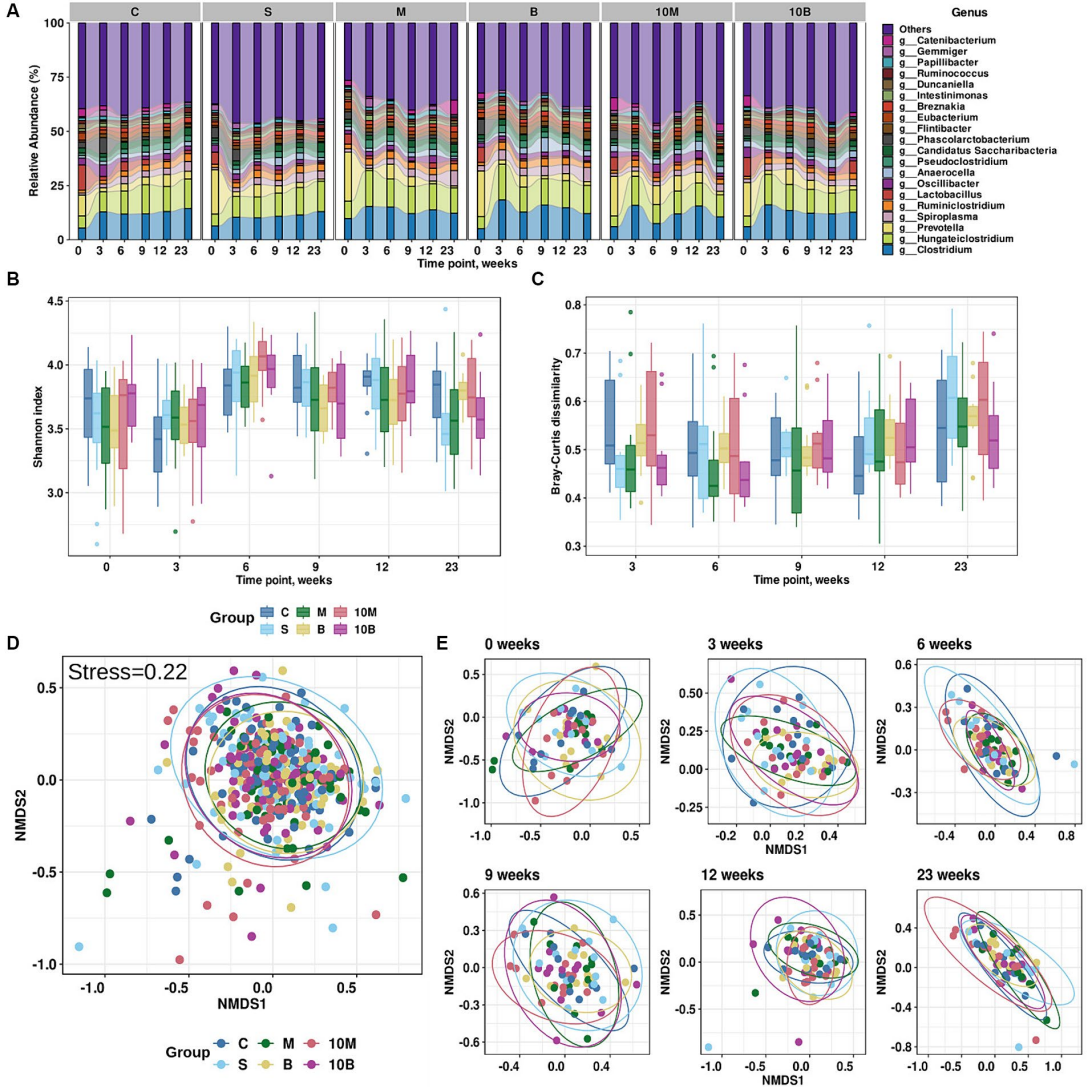
Results of exploratory analysis of gut microbiota changes in experimental groups. **(A)** Taxonomic distribution at the genus level for each experimental group across time points (weeks). X-axis indicates time points (weeks), while Y-axis shows relative abundance distributions in experimental groups. Different colors correspond to different taxonomic annotations. **(B)** Changes in Shannon index values for each experimental group across time points. X-axis indicates time points (weeks), Y-axis Shannon index values. Different colors correspond to different experimental groups. **(C)** Changes in Bray–Curtis dissimilarity calculated from baseline for each experimental group at different time points. X-axis indicates time points (weeks), Y-axis Shannon index values. Different colors correspond to different experimental groups. **(D)** Non-metric multidimensional scaling (NMDS) visualization of gut microbiome taxonomic profiles and Bray–Curtis dissimilarity metric (stress = 0.22). Different colors indicate different experimental groups. **(E)** NMDS visualization of **(D)** stratified by time point variable.

Sequencing identified 582 microbial species belonging to 265 total genera in 419 experimental rat’s fecal samples. Sample metadata is presented in Supplementary Table S1. After quality control, the fecal samples yielded 7,322 ± 2,506 reads per sample with length 1,470 ± 10 bp. A summary of the sequencing statistics is shown in Supplementary Table S2. Relative species abundances and taxonomic tables are presented in additional materials (Supplementary Tables S3, S4). The distribution of microbial genera by experimental group and time points are presented in Figure 1A.

According to variance analysis, microbiota richness, which can be explained by alpha-diversity, depends on the time point (Three-Way ANOVA with a Benjamini-Hochberg correction for multiple testing adj. *p* < 0.001), but does not depend on gender or experimental group. The Shannon index distribution by experimental group and time point is presented in Figure 1B. It is worth noting that the Bray–Curtis dissimilarity calculated between the baseline time point and subsequent ones was associated not only with the time point, but also with gender (Three-Way ANOVA with a Benjamini-Hochberg correction for multiple testing, adj. *p* < 0.001), while the experimental group was not a statistically significant variable. The distribution of Bray–Curtis dissimilarity from the baseline time point is presented in Figure 1C. However, according to variance analysis using permutations, the overall distribution of distances between fecal samples depended on the time point (*R*^2^ = 0.080, adj. *p* = 0.0003) and gender (*R*^2^ = 0.007, adj. *p* = 0.0003), but not on the experimental group (*R*^2^ = 0.013, adj.*p* = 0.06; PERMANOVA using the Bray–Curtis dissimilarity with a Benjamini-Hochberg correction for multiple testing; 9,999 permutations). On the other hand, analysis of multidimensional scaling and visual inspection did not reveal clear clusters into which the samples could be divided (Figures 1D,E).

**Figure 2:**
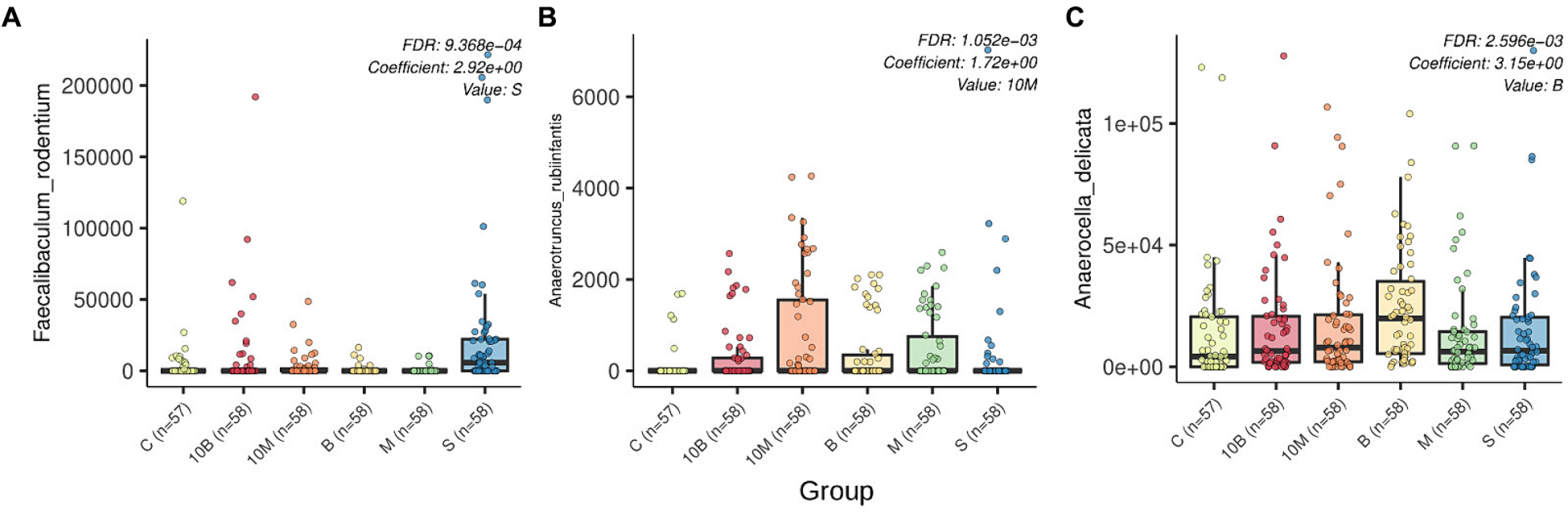
Box plots generated by MaAsLin2 show the differential abundance of microbial species by experimental group. FDR *p*-values and regression coefficients as well as experimental group variables are shown in the upper right corner of each plot. The analysis was performed with the following parameters: fixed_effects = c(“sex,” “group”), reference = c(c(“group, control”)), random_effects = c(“time_point,” “sourceid”), max_significance = 0.01. The x-axis indicates experimental group, while the y-axis indicates relative abundance. Differently abundant species are indicated by letters: **(A)** *Faecalibaculum rodentum*; **(B)** *Anaerotruncus rubiinfantis*; **(C)** *Anaerocella delicata*.

LefSe analysis was performed on the differential abundances of detected taxa linked to experimental groups. Our research data showed that in the control group where rats were treated with sucrose, the relative abundance of *Faecalibaculum genera* and *Faecalibaculum rodentium* in GM increased, while the SP-fed experimental groups were not associated with an increase in specific microorganism taxa (Supplementary Table S5). Multivariate analysis considering the influence of several factors on microbiota structure showed statistically significant associations of three bacterial species with different experimental groups. As with the LefSe analysis, rats from the sucrose-fed control group showed increases in *F. rodentium* according to MaAsLin2 results. In contrast to the LefSe analysis, MaAsLin 2 analysis identified additional associations such as increases in *Anaerutruncus rubiinfantis* in the experimental 10 ED_rat_ monellin-fed group. Interestingly, according to relative abundance visualization, *A. rubiinfantis* also increased in the 1 ED_rat_ monellin-fed group, but this result is not statistically reliable. *Anaerocella delicata* increased in the fetal samples of 1 ED_rat_ brazzein-fed rats. MaAsLin 2 analysis results are presented in Figure 2. The MaAsLin 2 analysis data presented in relation to the time points showed that these associations do not tend to increase steadily in relative abundances; rather, they are fluctuant in nature and do not go beyond the standard deviation (Supplementary Figure S1). Additionally, the influence of time point on rat microbiome structure was evaluated (Supplementary Table S6).

## Discussion

In this study we tested the effects of consuming recombinant monellin and brazzein on GM. We did not find any general structural changes in microbiota associated with sweet protein consumption. However, we detected changes in the comparative representation of individual bacterial species, such as *Faecalibaculum rodentium*, *Anaerutruncus rubiinfantis*, and *Anaerocella delicata*.

Our results showed an increase in *Faecalibaculum rodentium* when control group rats consumed sucrose. This is consistent with published reports that sucrose promotes the growth of *F. rodentium*, which displaces Th17-inducing microbiota (12). *F. rodentium* remodels retinoic acid signaling to control eosinophil-dependent homeostasis of the gut epithelium (30). Microbiome profiling identified *F. rodentium* as a key species involved in this regulation. In enterocytes, *F. rodentium* reduces the expression of retinoic acid-producing enzymes Adh1, Aldh1a1, and Rdh7, a reduction in retinoic acid signaling required to maintain specific populations of gut eosinophils. Eosinophils suppress the intraepithelial-lymphocyte-mediated production of interferon-γ, which regulates epithelial cell function (30). Microbiota-induced Th17 cells provide protection by regulating lipid absorption across the gut epithelium in an IL-17-dependent manner. Diet-induced loss of protective Th17 cells was mediated by the presence of sugar. Excluding sugar from a high-fat diet protected mice from obesity and metabolic syndrome in a manner dependent on commensal-specific Th17 cells (12).

The results showed that monellin has a minor effect on the composition of GM. It is worth noting some fluctuations in the relative abundance of *Anaerotruncus rubiinfantis* occurred when consuming monellin, depending on the time point of the experiment. The *A. rubiinfantis* strain was first discovered in 2016. This strictly anaerobic species forms gram-positive non-spore forming cocci, but there is currently no information about its functions within the host (31, 32).

Brazzein also induced some changes in GM. Bioinformatics analysis showed a shift in the relative abundance of *Anaerocella delicata*. *A. delicata* was isolated from animal farm waste in Japan (33). At this point, there is too little information to say how this strain’s enrichment might affect gut metagenome function.

The largest shift in bacterial composition is linked to the time point associated with changes in animal housing conditions, irrespective of the intake of target substances. Brazzein and monellin result in minor changes in the GM of rats. Recently, assessment of the same experimental animals’ morphological, physiological, biochemical, hematological, and behavioral characteristics allowed us to conclude that monellin and brazzein are safe and nontoxic for the mammalian organism (21). Therefore, changes in microbiota under the influence of SPs have a rather neutral character.

## Conclusion

The problem of obesity has been quite acute in recent years. In order to avoid obesity and related diseases, more and more people are looking for substitutes to sugars in other sweeteners. In our study we examined the effect of sweet proteins brazzein and monellin on the composition of GM. The results showed that consuming such proteins affected the GM of rats to a lesser extent than changing their housing conditions from quarantine to experimental conditions.

## Data availability statement

The datasets presented in this study can be found in online repositories. The names of the repository/repositories and accession number(s) can be found below: https://www.ncbi.nlm.nih.gov/, PRJNA1043060.

## Ethics statement

The animal study was approved by the study was conducted according to the guidelines of the Declaration of Helsinki and the Ethics Committee at Pharmbiomed Scientific Research Centre. The study was conducted in accordance with the local legislation and institutional requirements.

## Author contributions

VV: Methodology, Writing – original draft, Writing – review & editing. DB: Methodology, Writing – review & editing. EO: Investigation, Writing – review & editing. KK: Conceptualization, Writing – original draft, Writing – review & editing. VB: Methodology, Writing – review & editing. NZ: Investigation, Writing – review & editing. AL: Methodology, Writing – review & editing. MM: Investigation, Writing – review & editing. PZ: Methodology, Writing – review & editing. PS: Conceptualization, Methodology, Writing – review & editing. OD: Conceptualization, Writing – review & editing. IM: Methodology, Writing – review & editing. TN: Conceptualization, Writing – original draft, Writing – review & editing. AK: Writing – original draft, Writing – review & editing. VL: Conceptualization, Writing – original draft, Writing – review & editing. ML: Conceptualization, Writing – original draft, Writing – review & editing.

## Footnotes

^1^ https://github.com/rrwick/Porechop
