# Supplementary figures and images for: Effect of the consumption of brazzein and monellin, two recombinant sweet-tasting proteins, on rat gut microbiota
Source: Front Nutr. 2024 Mar 21;11:1362529. doi: 10.3389/fnut.2024.1362529 (PMC10991754; doi:10.3389/fnut.2024.1362529)

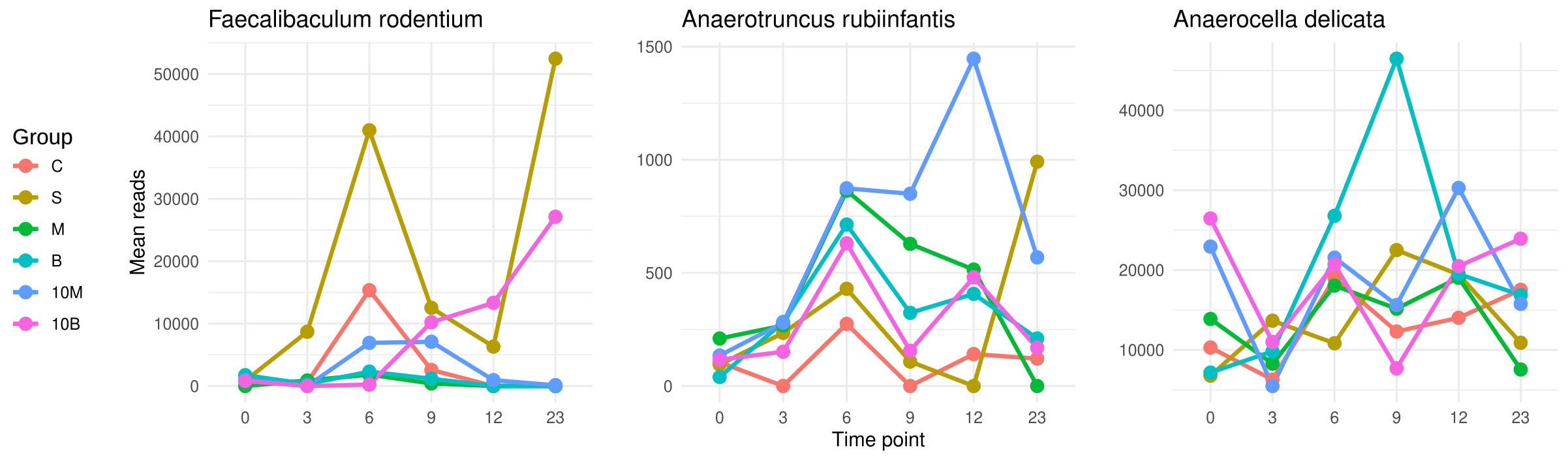

Supplement: Supplementary file 1 [file Image_1.TIFF]
